# The use of intravesical BCG in urothelial carcinoma of the bladder

**DOI:** 10.3332/ecancer.2019.905

**Published:** 2019-02-26

**Authors:** Omar Alhunaidi, Alexandre R Zlotta

**Affiliations:** 1 Department of Surgical Oncology, Division of Urology, University of Toronto and University Health Network, Toronto, ON M5G 1L7, Canada; 2 Department of Surgery, Division of Urology, Al-Amiri Hospital, Kuwait City, PO Box 4077, Safat 13041, Kuwait; 3 Department of Surgery (Urology), Mount Sinai Hospital, Toronto, ON M5G 1X5, Canada; 4 Lunenfeld-Tanenbaum Research Institute, Mount Sinai Hospital, Toronto, ON M5G 1X5, Canada

**Keywords:** bladder cancer, bacillus Calmette–Guérin, non-muscle invasive bladder cancer, urothelial carcinoma, intravesical therapy

## Abstract

The high recurrence and progression rates of non-muscle invasive bladder cancer (NMIBC) have led investigators to study the use of intravesical therapy in order to prevent them. Bacillus Calmette–Guérin (BCG) has been successfully used for this indication to treat NMIBC for more than four decades.

BCG is the only intravesical agent shown to reduce the risk of progression of NMIBC to muscle-invasive disease. Despite over 40 years of clinical use, the precise mechanism of action for what has often been considered the most successful cancer immunotherapy in humans remains largely unknown.

Unfortunately, BCG therapy is not a universal panacea and it still fails in up to 40% of patients. Many of these patients, especially in the high-risk category (T1 high-grade disease, carcinoma in situ) will require aggressive therapy like cystectomy or in selected cases, bladder-sparing options like chemo-radiation. Indeed, there is no gold standard intravesical treatment after BCG failure.

## History of BCG

Bacillus Calmette–Guérin (BCG, Mycobacterium *bovis*) was first discovered as a vaccine against tuberculosis by French scientists Albert Calmette and Camille Guerin in 1921 at the Pasteur Institute in Lille, France and it was named after them [1]. Intrigued by the hypothesis of Pearl in 1929, that clinical tuberculosis may cause lower frequency of tumours in autopsy materials, in the 1950s, Old, Clark and Benacerraf observed that BCG also prevented the growth of experimental tumours [2].

Mathe *et al* [3] in 1969 showed that BCG has an effect against human leukaemia. Morton *et al* [4] in 1970 demonstrated that intralesional BCG has an effect against human melanoma. Zbar *et al* [5, 6] described the principal rules for adequate immunotherapy.

- Tumour burden must be small.
- Direct contact between BCG and tumour is essential.
- The dose of the immunising agent must be adequate.
- Tumours respond better when confined to the parent organ or, in case of metastases, when only in regional lymph nodes.

Bloomberg *et al* [7] in 1975 showed that local BCG causes strong inflammatory reactions in the healthy bladder of dogs. Morales in Canada was then the first to use BCG vaccine in the bladder for the treatment of recurrent non-muscle invasive (previously named ‘superficial’) bladder cancer (NMIBC) in 1976 [8]. He reported the use of this regimen on 10 patients (7 only eligible for analysis) with recurrent ‘superficial bladder cancer’. He instilled 120 mg of BCG (Frappier strain, Montreal) packaged in vials of 6, in 50 cc of saline via a urethral catheter into the bladder. The treatment regimen was weekly instillations intravesically of BCG for 6 weeks after initial intradermal injection of BCG to ‘prime’ patients. Morales noted that at least 3–6 weeks were needed to mount delayed hypersensitivity reaction and side effects lasted 1 week. Favourable outcomes on recurrence were reported in this very limited series [8].

In 1980, Lamm and colleagues completed the first randomised controlled trial proving the clinical effect of BCG on NMIBC [9]. In 1982, Brosman *et al* [10] modified the Morales intravesical BCG regimen and discontinued the intradermal injections. BCG was approved by the Food and Drug Administration in 1990 for the treatment of carcinoma in situ (CIS) of the bladder [11].

## Mechanism of action

The mechanism of action of BCG is very complex. In a nutshell, it is likely that BCG antitumor immunotherapy is the result of the immune response initially aimed at clearing the foreign pathogen instilled in the bladder. The end result is an activated immune response that is needed to combat the attenuated mycobacteria which are necessary to target tumour cells [12–15].

Our own work and a review by Redelman-Sidi *et al* [16] briefly summarise the different steps [17]. The first step is the control against mycobacteria and the second step is the control against tumour cells. BCG first needs to attach to the urothelium through Fibronectin and Integrins. The Antigen 85 complex of mycobacteria, including BCG, plays a central role in synthesising major components of the inner and outer leaflets of the mycobacterial outer membrane and binds Fibronectin. BCG attaches to the urothelium through Fibronectin [17, 18]. BCG is then internalised by urothelial cells and captured by the first line of innate immune response cells. Antigen presentation and cytokine release result in major histocompatibility complex (MHC) II upregulation and of IL-6, Il-8 and granulocyte-macrophage colony-stimulating factor (GM-CSF). Immune cells are then recruited to the ‘war zone,’ including granulocytes, CD4 and CD8 T cells, natural killer (NK) cells and macrophages. A torrent of mainly TH-1 cytokines, including Interferon gamma, IL-1, IL-12, IL-18, IL-23 and tumour necrosis factor (TNF)-alpha are produced by these immune cells.

This response is mainly non-specific although we have shown that a major increase in the lymphoproliferative response against purified protein derivative, Antigen 85. BCG culture filtrate and whole BCG can be observed in the peripheral blood of patients with NMIBC treated with BCG, suggesting specific immune responses against numerous BCG subfractions [17]. Local immune responses are exemplified by the granulomas, which can be observed in the bladder wall of patients treated with BCG.

Bladder tumour cell killing involves an immune-mediated cytotoxicity, including NK cells, NK T cells, CD8 T cells, macrophages and TRAIL (granulocytes) among many others. We have previously investigated the immunologically active components of BCG in the therapy of bladder cancer [19]. We observed that numerous BCG subcomponents, including BCG cell wall, plasma membrane, cytosol, purified polysaccharides as glucan or arabinomannan, purified native proteins from BCG culture filtrate, phosphate transporter PstS-2 and -3 proteins provide positive stimuli for Th1 cell differentiation and enhance the cytotoxicity against bladder tumour cells.

As redundancy is a keyword in biology, it seems that the immunological complexity of live attenuated mycobacteria is necessary to trigger the avalanche of immune responses required to clear and control tumour cells locally in the bladder. Response to BCG therapy, or the absence of response, is driven by a multitude of parameters. One of the most common strategies is the activation of innate immune pathways.

Toll-like receptor (TLR) pathways are of particular interest in cancer immunotherapy. TLRs are a family of receptors that bind to common components of many pathogens, as well as signals released by damaged cells. TLRs are expressed on many innate immune cells, including dendritic cells. Dendritic cells, the most potent of all antigen presenting cells, play a pivotal role in bridging the innate and adaptive responses. Thus, targeting dendritic cell TLRs is a common strategy for enhancing adaptive immune responses. TLRs are also present on a large portion of bladder tumours where higher TLR expression is correlated with less invasive tumours and expression levels of this important receptor may drive antitumor response [20]. Some individuals show a higher level of natural resistance than others to infection with certain intracellular pathogens, including BCG.

The well-known gene encoding natural resistance-associated protein 1 (*NRAMP* 1) exists in two allelic forms, differing by a point mutation. Polymorphisms in *NRAMP1* and *hGPX1*gene to BCG have resulted in decreased cancer-specific survival (CSS) for the *NRAMP1 D543N* G:G genotype, as well as reduced recurrence-free survival (RFS) and increased risk of recurrence post-BCG [21].

## BCG strains: is there a difference?

There have been several strains of BCG developed since the original strain in 1921, but whether these strains have varying efficacies on bladder tumours remain unclear. While hundreds of thousands of patients have been treated with BCG for prevention of NMIBC, no clinical difference has been shown among studies despite the use of various strains worldwide.

Trials using various strains have consistently demonstrated the efficacy of BCG immunotherapy in reducing recurrence and progression of NMBIC in all countries across the globe. BCG is recommended by all scientific associations from the European Association of Urology (EAU), American Urological Association, Japanese Association or the Canadian Urological Association.

Sengiku and colleagues demonstrated no significant difference between the studied strains [22]. Supporting a previous European Organization for Research and Treatment of Cancer (EORTC) meta-analysis which suggested that there is no large difference between different strains [23], a recent meta-analysis of randomised trials performed by Quan *et al* [24] concluded that no meaningful correlations between BCG strain and other survival outcomes (RFS, CSS and overall survival) could be drawn. Another systematic review and meta-analysis by Chou *et al* [25] concluded that no comment can be made regarding differences among strains. Gan *et al* [26] studied the effects of substrain differences in BCG immunotherapy for bladder cancer and came to a similar conclusion.

The only randomised study to have suggested some differences between different BCG strains was reported by Rentsch *et al* [27], who compared the clinical efficacy, immunogenicity and genetics of the BCG Connaught and Tice strains. Treatment with BCG Connaught conferred significantly greater 5-year RFS compared with treatment with BCG Tice (*p* = 0.0108). No statistically significant difference was observed for progression-free survival. Despite being randomised, this study had some limitations. For instance, an uneven number of patients were included in each arm despite a relatively small number of patients overall [27].

Interestingly enough, a vaccination for tuberculosis, which affects billions of humans worldwide and has been extensively studied, has shown significant differences in the immune response induced by different BCG vaccine strains from animal and human studies. The key question remains whether these differences *in vitro* translate to differences in protective efficacy against tuberculosis in humans, as well as in bladder cancer patients. What must be kept in mind is that the doses for vaccination and intravesical therapy are dramatically different [28].

## Intravesical BCG dose and schedule

To obtain the standard dose, the BCG vaccine powdered vial is usually diluted into 50 ml of normal saline. The diluted BCG is then infused into the bladder through a urethral catheter after complete drainage of the bladder. It should be maintained in the bladder for 2 hours. BCG is administered for 2 to 4 weeks after resection to prevent the risk of systemic toxicity [29]. The schedule of intravesical BCG treatment comprises an induction course (6 weekly treatments) and a maintenance course [8, 30].

We have previously shown that in most patients, the maximal peripheral immune response is already observed after 4 weekly BCG instillations. However, patients not previously immunised against mycobacterial antigens may require 6 weekly instillations to achieve a maximum stimulation level [30]. Following the induction course, several studies have reported that additional BCG treatment may decrease recurrence.

Two decades ago, Zlotta *et al* [17] showed that intravesical BCG instillations induced a transient (less than 6 months) peripheral immune activation against BCG antigens. Reactivation was observed in most cases after additional BCG courses [17]. This absence of long-lasting immune activation after a single 6-week course of BCG could be related to the increased clinical efficacy observed with BCG maintenance instillations. However, the optimum period of BCG maintenance is still controversial. The Southwest Oncology Group BCG maintenance regimen was a weekly dose for 3 weeks at 3, 6, 12, 18, 24, 30 and 36 months [13, 31, 32].

The EORTC–Genitourinary (GU) group compared 1-year maintenance therapy versus 3-years maintenance therapy in intermediate and high-risk patients [33]. This study showed that intermediate risk patients should have a maintenance course for 1 year as there was no further improvement in outcome by the extended 3 years course. However, for high-risk patients, the 3 years regimen was superior in reducing the recurrences compared to the 1-year course [33]. This trial also compared a full standard BCG dose to one-third BCG dose. It did not show any difference in toxicity between full dose and one-third of the standard BCG dose, but the one-third dose was associated with increased rates of recurrences as it was a suboptimal dose [33]. More recently, the Club Urológico Español de Tratamiento Oncológico showed in a randomised trial that a maintenance course with a single BCG dose every 3 months for 3 years is superior to induction alone in high-risk patients with NMIBC [34]. The EAU guidelines recommend BCG induction therapy, as well as 1 year of maintenance BCG therapy for the intermediate risk group. For the high-risk group, the recommendation is 1 to 3 years of maintenance BCG therapy [32].

Meta-analyses suggest a more balanced view. Indeed, Quan *et al* [24] combining over 2,000 patients from various randomised trials did not observe any difference between low and standard dose. Similarly, Chou *et al* [25] concluded that head-to-head trials showed no clear differences between standard and lower doses of BCG in risk of recurrence, progression or mortality, including among patients with higher-risk NMIBC. There was some inconsistency though with standards of evidence deemed low.

## Prevention of recurrence

Shelley and colleagues in a meta-analysis showed that BCG therapy was superior to resection alone for prevention of NMIBC recurrences [35]. Several meta-analyses have proven the superiority of BCG after resection compared to resection alone or compared with intravesical chemotherapy in preventing recurrences [36, 37]. A meta-analysis by Malmström and colleagues showed that intravesical BCG with maintenance course has 32% reduction in risk of recurrence compared to Mitomycin C (MMC) intravesical chemotherapy [38]. In a randomised phase 3 study 30911, the EORTC-GU group compared epirubicin, BCG and BCG plus isoniazid in intermediate- and high-risk NMIBC [39]. Intravesical BCG with or without isoniazid was superior to epirubicin for preventing recurrence in both intermediate and high-risk groups [39]. Duchek *et al* [40] compared BCG alone to epirubicin and interferon-alpha2b in a multi-centric randomised control trial. Patients were given an induction course, followed by a maintenance course for 2 years. BCG was superior in preventing recurrence compared to the combination of epirubicin and interferon-alpha2b [40]. Another randomised controlled trial assessed the long-term efficacy of maintenance BCG compared to maintenance MMC in recurrent superficial bladder tumours with 20 years follow up. BCG significantly reduced recurrences compared to MMC [41].

## BCG effect on progression

BCG has been consistently shown to decrease the progression rates of NMIBC. A meta-analysis by Sylvester and colleagues outlined the benefits of BCG maintenance in preventing progression of NMIBC although interestingly enough this benefit was limited to CIS only [23]. Another meta-analysis by Bohle and colleagues demonstrated statistically significant superiority of BCG with maintenance course compared to MMC maintenance for reducing the risk of progression in superficial bladder tumours [42].

## BCG failure and subclassification

About 40% of patients of NMIBC will fail intravesical BCG treatment. Although many factors might lead to BCG failure, the dose of BCG and type of T helper response (Th1 or TH2) may lead to dramatically diverging outcomes. Low-dose BCG might not trigger enough TH1-type immune response, which is the main response to BCG activity. Too high doses of BCG may paradoxically activate mixed TH1/TH2 responses which will counterbalance the TH1 response [15, 43]. Other factors of BCG failure include occult micrometastatic disease prior to BCG therapy [15].

Patients who fail intravesical BCG treatment are usually sub-classified into three categories based on the type of failure:

- BCG refractory, which is the persistence of disease after induction or maintenance BCG treatment.
- BCG relapse, the recurrence of disease after a disease-free period post BCG treatment.
- BCG intolerance when the patient is not tolerating the completion of BCG induction [44].

The definitions, endpoints and clinical trial designs for NMIBC as recommended by the International Bladder Cancer Group might serve as an excellent current state-of-the-art resource [45]. The type of failure (BCG unresponsive, refractory, relapsing or intolerant) should be clearly defined. Because stakes are very high for these patients, for whom BCG has failed, and options are limited, single-arm designs may be relevant for the BCG-unresponsive population. The consensus for a clinically meaningful initial complete response rate (for CIS) or recurrence-free rate (for papillary tumours) is of at least 50% at 6 months, 30% at 12 months and 25% at 18 months.

Despite promising studies, there is no current gold standard intravesical after BCG failure. Radical cystectomy (and trimodal therapy for selected patients) remains the standard treatment to prevent further disease progression [32, 46]. However, the future seems brighter. Given the efficacy, approval and increased use of immune checkpoint inhibitors against PD-L1 or PD-1 for advanced and metastatic bladder cancer, or platinum—ineligible or resistant, the role of these agents in BCG-relapsing disease at various disease stages is under consideration. Current trials in BCG-unresponsive disease are underway, including the NCT02808143 (https://clinicaltrials.gov/ct2/show/NCT02808143) trial where Pembrolizumab is administered intravesically in combination with BCG in patients with high risk or BCG-refractory NMIBC.

The NCT02844816 trial evaluates the complete response at 25 weeks after intravenous Atezolizumab (https://clinicaltrials.gov/ct2/show/NCT02844816) in patients with a CIS component and the event-free survival at 18 months in patients with BCG-unresponsive high-risk NMIBC (Ta/T1/CIS). These trials may prove difficult to complete, as shown by the Nivolumab trial NCT03106610 in BCG failure which was terminated because of slow accrual (https://clinicaltrials.gov/ct2/show/NCT03106610).

A number of randomised controlled trials have studied the use of Electromotive Drug Administration (EMDA) of intravesical MMC in NMIBC but evidence remains lacking on the use of EMDA-MMC in patients who have become unresponsive to BCG therapy. Our group has recently reported our early data, albeit in a very limited series of 30 patients, showing evidence of progression-free survival in over 60% of patients at the 2-year mark [47].

## Conclusions

BCG remains the gold standard treatment for patients with intermediate- and high-risk NMIBC. After four decades of use, the exact mechanism of action remains unknown and further studies would be helpful to augment its efficacy. However, the future of BCG for preventing recurrences and progression in NMIBC looks still bright as no other effective therapy for these patients appears on the horizon.

The space of BCG failure is the object of intense research activity as currently there is no gold standard intravesical treatment. Hopefully, new intravesical therapies or immune checkpoint blockade agents will meet this unmet need for patients and their physicians.

## Conflicts of interest

None.

## Funding statement

No source of funding was given for this review.
